# Supplementary material for: Heterodyne analysis of high-order partial waves in attosecond photoionization of helium
Source: Nat Commun. 2025 Jan 3;16:381. doi: 10.1038/s41467-024-55247-1 (PMC11698836; doi:10.1038/s41467-024-55247-1)
Supplement: Supplementary file 1 — Supplementary Information [file 41467_2024_55247_MOESM1_ESM.pdf]

Supplementary Information for
Heterodyne analysis of high-order partial waves in attosecond photoionization of helium

Wenyu Jiang[1,2,*], Luke Roantree[3,*], Lulu Han[1,*], Jiabao Ji[4], Yidan Xu[1], Zitan Zuo[1], Hans Jakob Wörner[4], Kiyoshi Ueda[1,5,6], Andrew C. Brown[3,†], Hugo W. van der Hart[3], Xiaochun Gong[1], Jian Wu[1,7,8,†]

[1]*State Key Laboratory of Precision Spectroscopy, East China Normal University, Shanghai 200241, China*
[2]*Present address: School of Physics and Microelectronics, Zhengzhou University, Zhengzhou 450001, China*
[3]*Centre for Light-Matter Interaction, School of Mathematics and Physics, Queen's University Belfast, Northern Ireland, BT7 1NN United Kingdom*
[4]*Laboratorium für Physikalische Chemie, ETH Zürich, 8093 Zürich, Switzerland*
[5]*Department of Chemistry, Tohoku University, Sendai 980-8578, Japan*
[6]*School Physical Science and Technology, ShanghaiTech University, Shanghai 201210, China*
[7]*Collaborative Innovation Center of Extreme Optics, Shanxi University, Taiyuan, Shanxi 030006, China*
[8]*Chongqing Key Laboratory of Precision Optics, Chongqing Institute of East China Normal University, Chongqing 401121, China*
∗ *These authors contribute equally to this work.*
† *e-mail: andrew.brown@qub.ac.uk, jwu@phy.ecnu.edu.cn*

Contents

Supplementary Note 1: Available higher-order transitions

Supplementary Note 2: Extracting the anisotropy parameters from photoelectron angular distributions

Supplementary Note 3: Comparison of delay-resolved anisotropy parameters

Supplementary Note 4: Combined contribution of component signals

Supplementary Note 1: Available higher-order transitions

Due to the $1s^2$ ground state of neutral helium, the two-photon transitions available in a typical RABBITT scheme permit only ϵs or ϵd_0 final states (where we use ϵ to denote 'continuum' states). Upon extending RABBITT to include four-photon transitions, the ϵg_0 final state may also be populated. To reach higher electron angular momenta, at least 6-photon transitions are required. The R-Matrix with Time-dependence (RMT) approach allows the photoelectron spectra to be calculated using only specified partial waves; we use this functionality to extract angle-resolved 2ω phase from three RABBITT spectra obtained using ($s-$, d_0-waves), ($s-$, d_0-, $g-$waves) and ($s-$, d_0-, g_0-, i_0-waves). Supplementary Figure 1(a) demonstrates that the inclusion of the i_0-wave, the first sideband-accessible partial-wave beyond the g_0-wave, induces almost no change to the angle-resolved 2ω phase as compared with a calculation comprising only the $s-$, d_0- and g_0-waves. At the IR intensity used in this investigation (1×10^{12} W/cm^2), the $s-$ and d_0-waves alone are shown to be insufficient to describe the angular behavior of the phase– in particular around the angular interval between $30° - 60°$. Supplementary Figure 1(b) provides further clarity on the level of agreement achieved by the (s, d_0) and (s, d_0, g_0) descriptions with a more complete (s, d_0, g_0, i_0) description. The maximum and mean absolute differences of the (s, d_0, g_0, i_0) description relative to the (s, d_0) description are 0.919 radians and 0.218 radians respectively, while the maximum and mean differences calculated relative to the (s, d_0, g_0) description are only 0.085 radians and 0.0160 radians respectively. If only the angular regions which can be reliably measured experimentally are considered, as indicated by the shaded region in Supplementary Figure 1(b), the maximum and mean absolute differences in 2ω phase between the (s, d_0, g_0) and (s, d_0, g_0, i_0) descriptions drop to only 0.030 radians and 0.011 radians respectively.

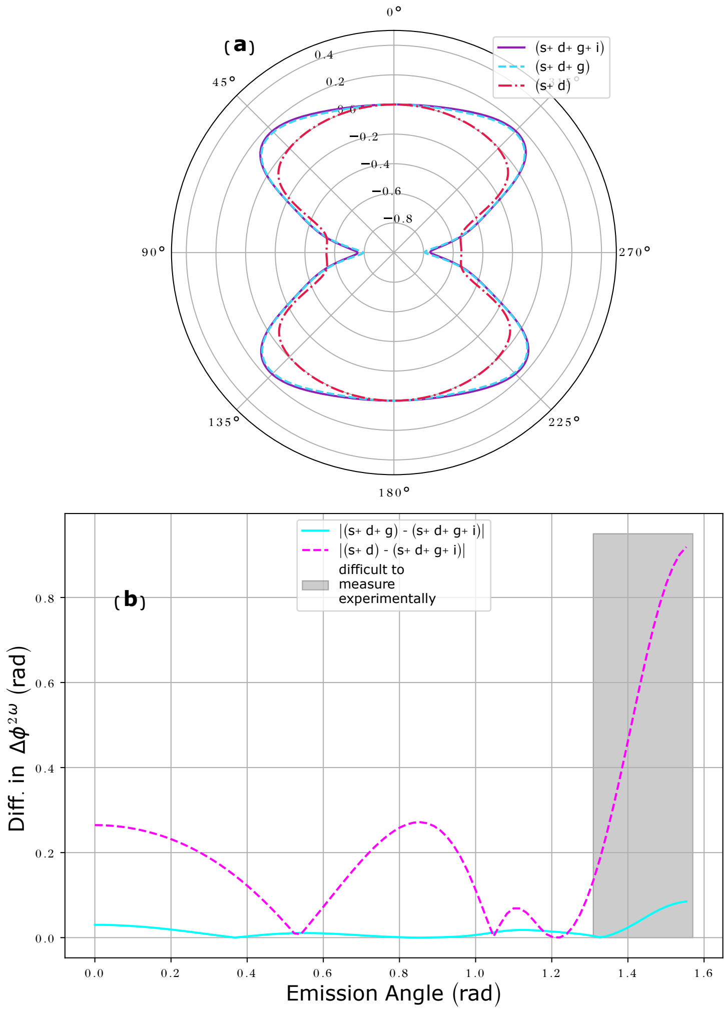

Supplementary Figure 1: Influence of high-order transitions on the angle-resolved relative phase shifts in SB16.(**a**) $\Delta\phi_{\rm rel}^{\rm SB16}(\theta)$, in units of $\pi-$radians, extracted across SB16 (from simulation) using only ($s-$ and d_0-waves) [red, dot-dashed], ($s-,\ d_0-$ and g_0-waves) [blue, dashed], ($s-,\ d_0-,\ g_0-$ and $i-$waves) [purple, solid]. (**b**) Absolute difference in $\Delta\phi_{\rm rel}^{\rm SB}(\theta)$, over the emission angle interval $[0, \pi/2]$, between ($s-,\ d_0-$ and g_0-waves) and ($s-,\ d_0-,\ g_0-$ and i_0-waves) [blue, solid], and between ($s-$ and d_0-waves) and ($s_0-,\ d_0-,\ g_0-$ and i_0-waves) [pink, dashed]. In (**b**), the angular interval in which there is most difficulty resolving experimentally is shaded.

Supplementary Note 2: Extracting the anisotropy parameters from photoelectron angular distributions

In a system such as atomic helium, where the singly-ionized electrons may be coupled to only a single ionic state, the lowest-order paths to the continuum require absorption of one XUV photon. This forms the mainbands and changes the system's orbital angular momentum by 1– in the case of helium 1-photon transitions to mainbands lead to p_0-waves only. As all further NIR-induced transitions will then start from p_0-waves, the sideband photoelectrons can be expressed in terms of only even Legendre polynomials:

$$I(\theta,\tau) \propto \sum_{n=0,2,4,6} \beta_n(\tau) P_n[\cos(\theta)], \tag{S1}$$

The pump-probe time delay resolved anisotropy parameters are extracted by projecting the experimental and theoretical photoelectron angular distributions onto the even-order Legendre polynomials [1–6]. These anisotropy parameters are, under the restriction of parallel linear polarization of the laser fields, scalar multiples of the spherical harmonics comprising the observed angle-resolved sideband signal. Which spherical harmonics comprise this signal is determined by interference between partial waves; and so we may expect to observe different anisotropy parameters for different component signals of the sideband as they originate from different interferences. The angular distribution of signal, $W(l_1, l_2)$, resulting from interference between a pair of partial waves with angular momenta l_1 and l_2 is given by the product of their associated spherical harmonics ($Y_{l_1,0}(\theta)$ and $Y_{l_2,0}(\theta)$), which may itself be expressed on the basis of spherical harmonics;

$$\begin{aligned} W(l_1,l_2) =& Y_{l_1,0}(\theta)\, Y_{l_2,0}(\theta)^* \\ =& \sum_{L=|l_1-l_2|}^{|l_1+l_2|} \sqrt{\frac{(2l_1+1)(2l_2+1)(2L+1)}{4\pi}} \begin{pmatrix} l_1 & l_2 & L \\ 0 & 0 & 0 \end{pmatrix}^2 Y_{L,0}(\theta)\,, \end{aligned} \tag{S2}$$

where $\begin{pmatrix} a & b & c \\ d & e & f \end{pmatrix}$ is a Wigner-3j symbol. We extract the strength of each $0\omega_{\mathrm{NIR}}$, $2\omega_{\mathrm{NIR}}$ and $4\omega_{\mathrm{NIR}}$ signal for each anisotropy parameter via a Fourier transform, mapping $\beta_n(\tau) \rightarrow \tilde{\beta}_n(\omega)$. Inspecting Equation S2, the highest order spherical harmonic (equivalently, anisotropy parameter) comprising interference between only s and d_0 waves is $Y_{4,0}(\theta)$ (corresponding to β_4). Observation of the non-zero β_6 parameter, particularly within the 2ω and 4ω component signals, provides direct evidence of the presence of higher-order partial-waves, and thus evidence of higher-order photoionization processes which cannot be satisfactorily explained by traditional (2-photon) RABBITT theory. In general RABBITT measurements in the perturbative regime, the partial-wave resolved two-photon phase shifts and proportions can be reconstructed by fitting the PADs and emission-angle resolved phase shift distributions with spherical harmonics [7,8]. However, with the introduction of the higher order processes, this kind of fitting becomes unstable due to the tiny proportion of the high-order partial waves– g_0- and i_0-waves here. In this case, the heterodyne analysis of anisotropy parameters serves as a sensitive tool to detect the participation of high-order partial waves.

Supplementary Note 3: Comparison of delay-resolved anisotropy parameters

Supplementary Figure 2 shows the $\beta_n(\tau)$ parameters extracted from simulated and experimental RABBITT spectra. The extremely small magnitude of the β_{10} coefficient in all cases (or, more importantly, its variation over time) indicates partial waves beyond the g_0-wave do not contribute significantly enough to be detected within the component signals. While the variation in β_8 is just large enough to be visible in Supplementary Figure 2(a) and (d), Equation S2 shows β_8 comprises interference between two g_0-waves, and interference between a d_0-wave and an i_0-wave. As each of these options requires interaction with the same number of photons, they both contribute to β_8 with a similar magnitude, making it difficult to attribute anything to a particular partial wave. In β_6, however, interference between g_0- and d_0 waves contributes significantly more than interference

between i_0- and s_0-waves, as $s-$ and $d-$ waves may both be reached via two-photon paths. As such, we limit our analysis of 'higher-order' anisotropy parameters to $\{\beta_0, \beta_2, \beta_4, \beta_6\}$.

By comparing the anisotropy coefficients in Supplementary Figure 2(b) and (c) (corresponding to only the s- and d_0-wave contributions from the 1 TW/cm^2 simulation and the 0.05 TW/cm^2 simulation), it is clear that while the signal contributions from s- and d_0-waves in the 1 TW/cm^2 case contain contributions from both 2- and 4-photon paths, the extracted anisotropy parameters show almost no difference from the 0.05 TW/cm^2 case where contribution from 4-photon paths may be considered negligible.

Further evidence of the relationship between the contribution of higher-order partial waves and the RABBITT phase is given from the experiment; comparing the magnitude of oscillations of the β_8 parameter (relative to the other β_n parameters) in Supplementary Figure 2(d) and (e), it is clear that the higher-order partial waves play an increasingly important role as the NIR intensity increases. The angular distributions of $2\omega_{\rm NIR}$ oscillation phase shifts of SB16 and SB18 with a NIR intensity of 3.2×10^{12} W/cm^2 are shown in Supplementary Figure 3(a) and (b).

From this, we conclude that the most significant differences to the photoelectron angular distribution (or its component signals) between 'standard' RABBITT and RABBITT-HOP may be attributed much more to interference with additional partial-waves than to any changes to the information contained in the phase of the s- or d_0-waves.

Supplementary Note 4: Combined contribution of component signals

It is well-known that when two component signals of the same frequency overlap, the resulting total signal has the same frequency. However, when there are relative differences in the component signals' phases and magnitudes, the calculation of the phase of the total signal is non-trivial. We

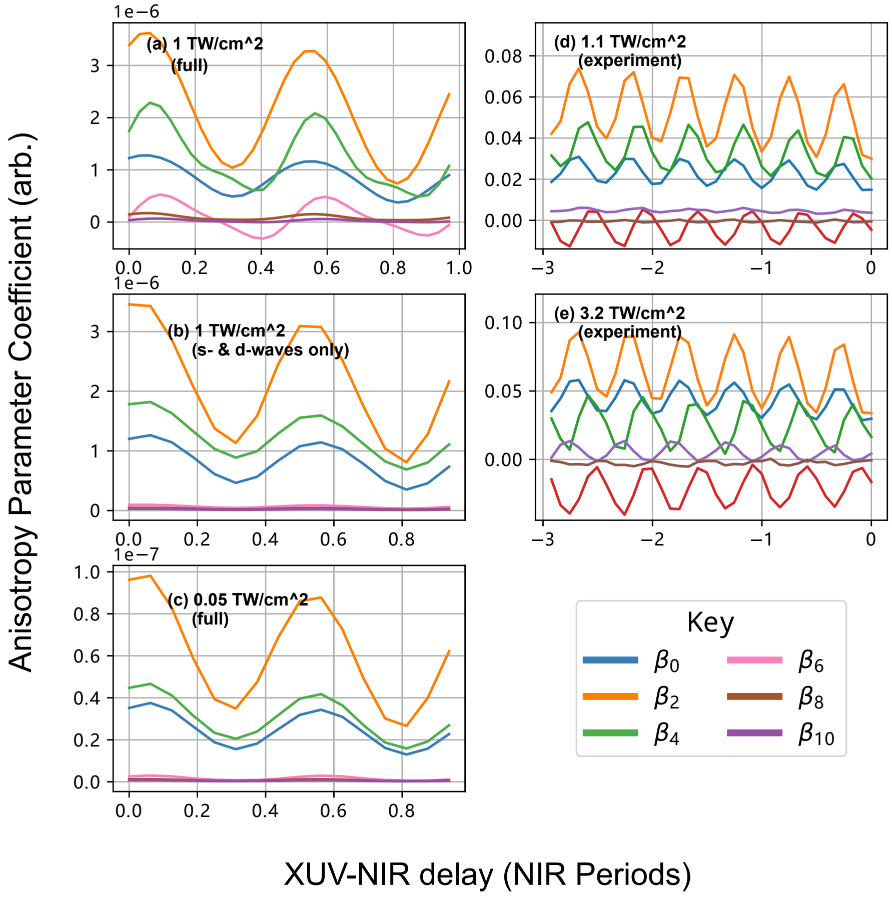

Supplementary Figure 2: Delay-resolved anisotropy coefficients. Delay-resolved anisotropy coefficients extracted from RABBITT scans from RMT simulation (**a-c**) and experiment (**d-e**). Subfigure (**a**) displays the anisotropy parameters extracted from the full output of an RMT simulation using a 1.0×10^{12} W/cm^2 NIR field, (**b**) the contribution of only $s-$ and d_0-waves to the output of an RMT simulation using a 1.0×10^{12} W/cm^2 NIR field and (**c**) anisotropy parameters extracted from the full output of an RMT simulation using a 5.0×10^{10} W/cm^2 NIR field. Subfigure (**d**) displays the anisotropy parameters extracted from an experiment using a 1.1×10^{12} W/cm^2 NIR field and (**e**) from an experiment using a 3.2×10^{12} W/cm^2 NIR field.

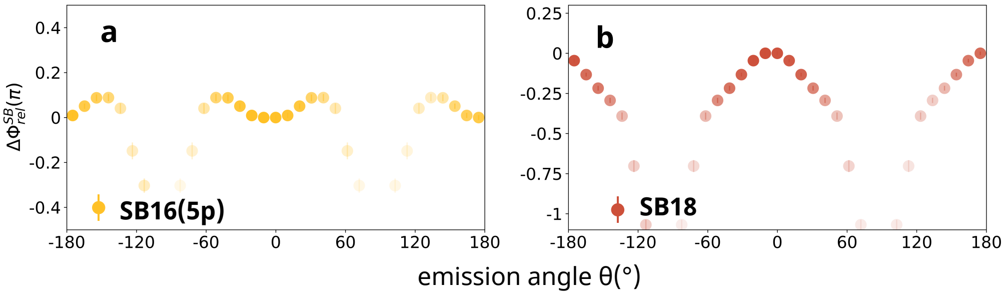

Supplementary Figure 3: The emission-angle-resolved $2\omega_{\mathrm{NIR}}$ phase shifts with a NIR field intensity of 3.2 TW/cm^2 as measured experimentally: (**a**) SB16($5p$), (**b**) SB18. The error bars represent the standard deviation.

show now that approximating the total signal's phase as the mean of the two-component phases, weighted by the component signal magnitudes, is accurate to second order in phase difference.

Let S be a total signal comprising two component signals with distinct magnitudes and phases. Without loss of generality, we will take the phase of the first component as zero – i.e. we describe the phase of the second component relative to that of the first.

$$S = A_1\cos(2\omega\tau) + A_2\cos(2\omega\tau + \phi)$$
$$= \left(\sqrt{A_1^2 + A_2^2 + 2A_1A_2\cos(\phi)}\right) \times \cos\left(2\omega\tau + \mathrm{atan}\left(\frac{A_2\sin(\phi)}{A_1 + A_2\cos(\phi)}\right)\right). \tag{S3}$$

Taylor expanding each term of S in terms of $\Delta\phi$ and truncating to second order, we obtain

$$S \simeq \left(\sqrt{A_1^2 + A_2^2 + 2A_1A_2}\right) \times \cos\left(2\omega\tau + \frac{A_2(\phi)}{A_1 + A_2}\right)$$
$$= (A_1 + A_2) \times \cos\left(2\omega\tau + \bar{\phi}\right), \tag{S4}$$

where $\bar{\phi}$ is the mean of the component signals' phases, weighted by their magnitudes.

Supplementary References

1. G. Laurent, W. Cao, H. Li, Z. Wang, I. Ben-Itzhak, C. L. Cocke, Attosecond control of orbital parity mix interferences and the relative phase of even and odd harmonics in an attosecond pulse train. *Physical Review Letters* **109**, 083001 (2012).

2. S. A. Aseyev, Y. Ni, L. J. Frasinski, H. G. Muller, M. J. Vrakking, Attosecond angle-resolved photoelectron spectroscopy. *Physical Review Letters* **91**, 223902 (2003).

3. J. Fuchs, N. Douguet, S. Donsa, F. Martin, J. Burgdorfer, L. Argenti, L. Cattaneo, U. Keller, Time Delays from One-Photon Transitions in the Continuum. *Optica* **7**, 154 (2020).

4. J. Peschel, D. Busto, M. Plach, M. Bertolino, M. Hoflund, S. Maclot, J. Vinbladh, H. Wikmark, F. Zapata, E. Lindroth, M. Gisselbrecht, J. M. Dahlström, A. L'Huillie, P. Eng-Johnsson, Attosecond dynamics of multi-channel single photon ionization. *Nature Communications* **13**, 5205 (2022).

5. J. Joseph, F. Holzmeier, D. Bresteau, C. Spezzani, T. Ruchon, J. F. Hergott, O. Tcherbakoff, P. D'Oliveira, J. C. Houver, D. Dowek, Angle-resolved studies of XUV-IR two-photon ionization in the RABBITT scheme. *Journal of Physics B: Atomic, Molecular and Optical Physics* **53**, 184007 (2020).

6. A. Boyer, V. Loriot, S. Nandi, F. Lépine, Probing Photoionization Dynamics in Acetylene with Angle-Resolved Attosecond Interferometry. *The Journal of Physical Chemistry A* **128**, 840–847 (2024).

7. W. Jiang, G. S. Armstrong, J. Tong, Y. Xu, Z. Zuo, J. Qiang, P. Lu, D. D. Clarke, J. Benda, A. Fleischer, H. Ni, K. Ueda, H. W. van der Hart, A. C. Brown, X. Gong, J. Wu, Atomic partial wave meter by attosecond coincidence metrology. *Nature Communications* **13**, 5072 (2022).

8. W. Jiang, G. S. Armstrong, L. Han, Y. Xu, Z. Zuo, J. Tong, P. Lu, J. M. Dahlström, K. Ueda, A. C. Brown, H. W. van der Hart, X. Gong, J. Wu, Resolving Quantum Interference Black Box through Attosecond Photoionization Spectroscopy. *Physical Review Letters* **131**, 203201 (2023).
